# Treatment intensification using long-acting insulin –predictors of future basal insulin supported oral therapy in the DIVE registry

**DOI:** 10.1186/s12902-015-0051-0

**Published:** 2015-10-07

**Authors:** Thomas Danne, Tobias Bluhmki, Jochen Seufert, Matthias Kaltheuner, Wolfgang Rathmann, Jan Beyersmann, Peter Bramlage, for the DIVE study group

**Affiliations:** Kinder- und Jugendkrankenhaus “AUF DER BULT”, Hannover, Germany; Institute of Statistics, University of Ulm, Ulm, Germany; Division of Endocrinology and Diabetology, Department of Internal Medicine II, University Hospital of Freiburg, Freiburg, Germany; Gemeinschaftspraxis Kaltheuner – v. Boxberg, Leverkusen, Germany; winDiab GmbH, Kehler Straße 24, 40468 Düsseldorf, Germany; Institut für Biometrie und Epidemiologie, Deutsches Diabetes Zentrum Düsseldorf, Düsseldorf, Germany; Institut für Pharmakologie und Präventive Medizin, Mahlow, Germany

**Keywords:** Oral antidiabetic drug, Insulin, Basal, Glycaemic control, Long-acting, Competing risks, Cox proportional hazards model

## Abstract

**Background:**

In patients with type-2 diabetes receiving oral antidiabetic drugs (OADs), the addition of insulin is frequently required to achieve sufficient control over blood glucose levels. It is, however, difficult to predict if, when and in which patients insulin therapy will be needed. We aimed to identify patient related variables associated with the addition of basal insulin to oral therapy resulting in a basal supported oral therapy (BOT).

**Methods:**

DIVE (DIabetes Versorgungs-Evaluation) is a prospective, observational, multi-centre diabetes registry established in Germany in 2011. For the present explorative analysis, 31,008 patients with type-2 diabetes prescribed at least one OAD were included. Patients who had previously received insulin and those over 90 years old were excluded. The event of interest was defined as the initiation of BOT during the observational period. Cause-specific Cox proportional hazards models based on a competing risk framework were applied for risk quantification.

**Results:**

Multivariable adjusted hazard ratios demonstrated that longer diabetes duration, higher BMI, poorer glycaemic control, documentation of any micro- or macrovascular comorbidity, the presence of concomitant non-antidiabetic pharmacotherapies, and greater numbers of prescribed OADs increased the likelihood of BOT initiation. On the other hand BOT initiation was less likely in patients with older age and female gender. Analysing the likelihood of OAD termination without initiation of BOT provided supportive evidence for the variables predictive of BOT initiation.

**Discussion:**

Analysis of the DIVE registry has resulted in the identification of a number of factors that may be predictive for the initiation of BOT for type-2 diabetes patients initially prescribed one or more OADs. Poor glycaemic control, the presence of vascular comorbidities and concomitant medications, and a greater number of OADs were all detected to increase the risk of a switch to BOT. Female gender and younger age showed protective properties.

**Conclusions:**

The close monitoring of patients displaying these characteristics may help to identify individuals who might benefit from early addition of insulin therapy to their oral treatment regimen.

**Electronic supplementary material:**

The online version of this article (doi:10.1186/s12902-015-0051-0) contains supplementary material, which is available to authorized users.

## Background

The first line treatment for patients with type-2 diabetes that cannot be controlled by diet alone is generally monotherapy with an oral antidiabetic drug (OAD) such as metformin. However, such treatment is rarely effective over an extended period of time [1]. Progressive decline in β-cell function along with decreasing insulin sensitivity generally necessitate the addition of other OADs, and eventually, insulin therapy is required in order to achieve adequate glucose control [2, 3].

Guidelines suggest that insulin therapy should be initiated when glycated haemoglobin (HbA1c) levels cannot be reduced below 7.5 % (58 mmol/mol) using OADs alone [4, 5]. A number of studies have demonstrated significant reductions in blood glucose levels with the addition of insulin to oral therapy [6, 7]. Moreover, the presence of OADs reduces the required dosage of insulin in comparison to when it is used alone [8]. There are three main options for the administration of insulin: conventional therapy (pre-mixed insulin), short-acting (prandial or supplementary), and long-acting (basal) therapy. While conventional therapy and short-acting insulin have been shown to achieve lower HbA1c levels, they are associated with weight gain and a higher risk of hypoglycaemia in comparison to the provision of a long-acting form [9]. The addition of basal insulin to an OAD regimen is termed basal supported oral therapy (BOT). The requirement for only a single injection per day is a great advantage of this treatment option, which is significant considering the reluctance of patients and clinicians alike to initiate insulin therapy [10–12].

There has been limited assessment of the characteristics of patients that convert to BOT. One study performed by Kostev et al. demonstrated that younger patients (<50 years) were more likely to switch to this treatment regimen [13]. They also found that BOT initiation was associated with shorter diabetes duration. The aim of the present analysis was to more clearly elucidate which factors could serve as predictors for the necessity to start BOT.

## Methods

### Study design

DIVE (DIabetes Versorgungs-Evaluation) is a prospective, observational, multi-centre diabetes registry established in Germany in 2011 [14]. Enrolment of patients started during the year 2011 with a retrospective capture of data back to January 1^st^ 2011. All patients enrolled for the registry provided written informed consent. Since then patients have been prospectively followed and the registry is still ongoing. For the purpose of data collection, the diabetes specific documentation software ‘DPV2 Diamax’ (Axaris Software and Systems GmbH, Germany) was used. The protocol received ethical approval from the Hannover Medical School Ethics Committee.

**Fig. 1:**
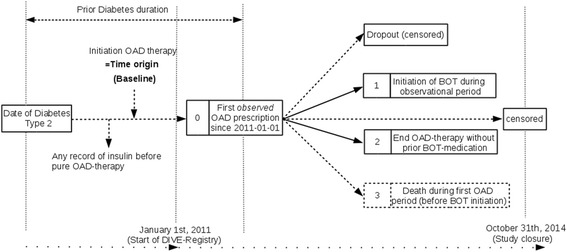
Competing risks model resulting from the *observable* data and including study-specific criteria. Time origin was set to OAD initiation. Any record of insulin use prior to baseline led to exclusion. Study entry (baseline) took place when firstly OAD prescription was recorded (state 0). State 1 represents BOT initiation, state 2 represents stopping OAD medication without BOT initiation being observed. Individuals with neither of these two events within the observational period were right-censored

**Fig. 2:**
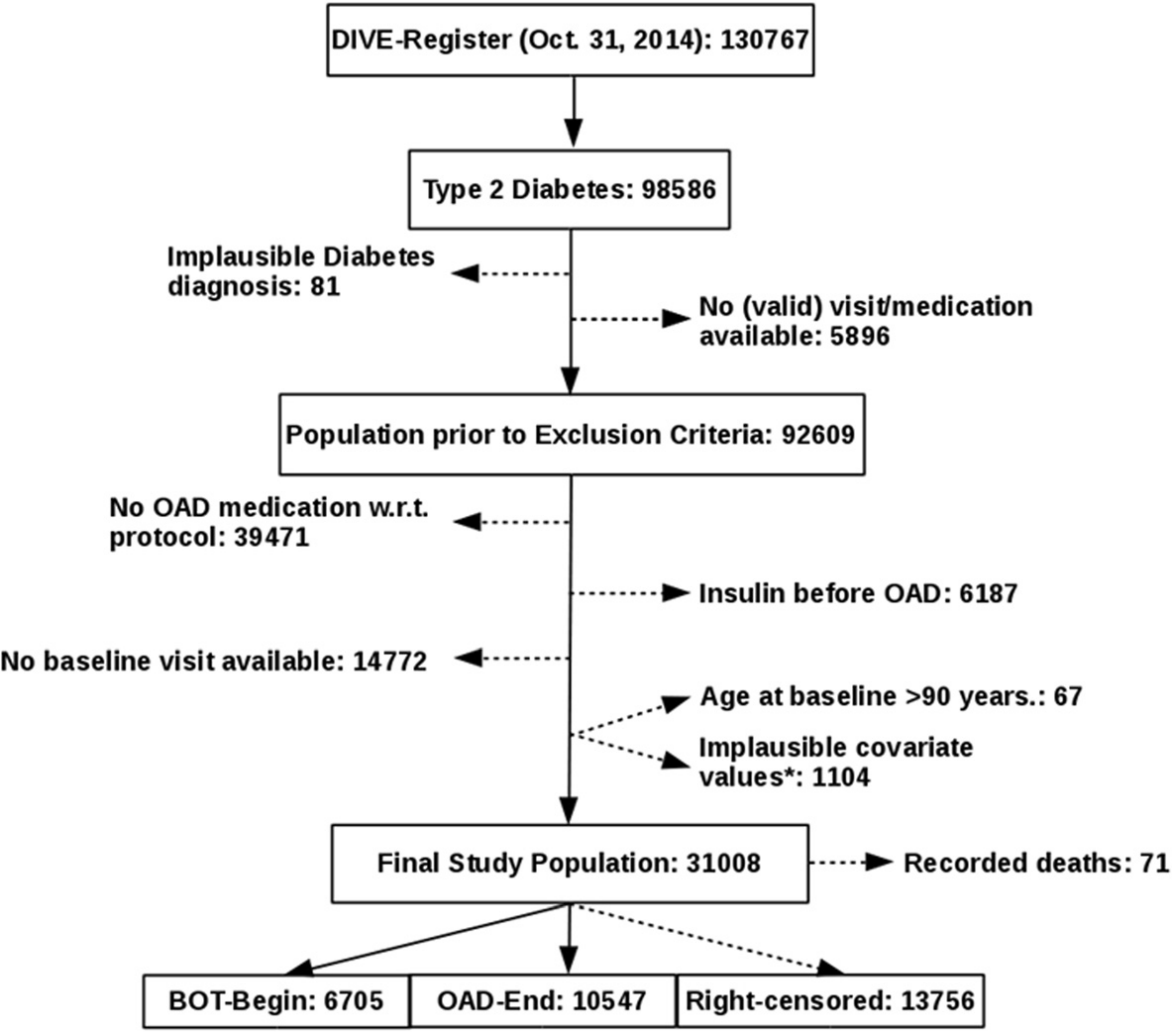
Patient flow chart. The final study population contained 31,008 individuals. Out of them, 6705 to BOT were observed. 24,303 patients did not initiate BOT within the observational period

For the current, explorative analysis, we considered only patients with type-2 diabetes and locked the data October 31^st^ 2014. Time origin was set to OAD initiation with at least one OAD (Fig. 1) that might have occurred even before start of DIVE registry was allocated to the first recorded OAD therapy since the start of DIVE-database. Patients already receiving any insulin therapy and patients older than 90 years were excluded (Figs. 1 and 2).

### Definitions of OADs and BOT used in the present study

The ATC index 2014 of the WHO Collaborating Centre for Drug Statistics and Statistics Methodology was used for classification of any drugs involved in the study (Additional file 1). OADs were defined as substances with an ATC index of A10B, excluding injectables such as human insulin and glucagon-like peptide-1 (GLP-1) agonists (A10BX04, A10BX07, and A10BX10). BOT was defined as the administration of one or more OADs in combination with the injection of long-acting insulin(s) (A10AE) and/or intermediate-acting neutral protamine Hagedorn (NPH)-insulin (A10AC01). Concomitant not diabetes related drugs were identified by ATC indices other than A10. Records of equal medications less than 6 months apart (e.g. metformin at baseline, missing documentation at one visit, metformin documentation at the next visit) were merged into one episode. Patients without further information regarding changes to their treatment were treated as dropouts 6 months after the last available medication record.

### Documentation

Exact dates of birth and of the diabetes diagnosis were not available. For this reason instead, time points were set as June 30^th^ of the corresponding year, or the 15^th^ of the corresponding month in order to enable the calculation of diabetes duration and patient age. Master data provided information about gender and diabetes type. Other patient characteristics at study entry including height, weight, number of OADs and concomitant medications, comorbidities, and laboratory values for fasting plasma glucose (FPG), postprandial plasma glucose (PPG), and HbA1c were derived from that doctoral visit, which was no more than 90 days prior to and 30 days after individual’s study entry. Individuals with no recorded visit within this time period were excluded from the study population (*n* = 14,772; Fig. 2). Relative HbA1c (%) was extracted from the available absolute HbA1c (mmol/mol) according to the following equation [15]:

$$ HbA1c\left(\%\right)=\frac{HbA1c\left(\frac{mmol}{mol}\right)}{10.929}+2.15. $$

The comorbidity profile of each patient was subdivided into (patient or physician reported) microvascular and macrovascular diseases within the year prior to study entry. The former included any record of retinopathy, blindness, nephropathy, renal failure, dialysis, or neuropathy. The latter includes transient ischaemic attack (TIA)/prolonged reversible ischaemic neurologic deficit (PRIND), stroke, coronary heart disease, myocardial infarction, and peripheral arterial disease. Similarly, (patient or physician reported) hypoglycaemia was defined as information about any severe hypoglycaemic event. Diabetes duration was computed as the time difference between diabetes diagnosis and study entry. At this stage, all variables were checked for plausibility.

### Statistics

To deal with missing data concerning diabetes duration, height, weight, FPG, and PPG (Additional file 2), the semi-parametric multiple imputation procedure of van Buuren, which is based on a chained equation approach, was applied [16] using the R-package *mi* [17]. Following the ‘missing at random’ (MAR) assumption, conditional regression models were built on all relevant baseline variables in addition to the individual outcomes. The imputed values for weight and height were used to calculate BMI. Analyses were conducted across ten imputed datasets. Pooled estimates were computed using Rubin’s Rule [18] and the R-package *mitools* [19]. Missing information about (severe) incidences like patient or physician reported hypoglycaemia and comorbidity profiles were assumed to be sufficiently reliable, i.e., treated as ‘not occurred’. Therefore, their imputation was not performed. PPG was also not imputed, since it was missing in 75 % of all individuals (Additional file 2: Table S2).

For descriptive purposes, mean differences (MDs) in continuous baseline covariates comparing the groups of patients with and without subsequent initiation of BOT (in the following named BOT(observed) and BOT(not observed)) were derived. Welch’s t-tests provided 95 % confidence intervals (CIs). Fisher’s exact test was used for categorical data. *P*-values of the corresponding odds ratios (OR) were presented. All descriptive statistics were based on available cases; i.e., they condition on observed future BOT outcomes.

Risks were expressed in terms of unadjusted and multivariable adjusted hazard ratios (HRs) with 95 % CIs obtained from Cox proportional hazards models regarding the event of interest ‘Initiation of BOT’ (state 1). All individuals initiating OAD therapy before study entry were handled as left-truncated data, i.e., they entered the risk sets associated with the Cox model at their individual times since start of OAD upon study entry. The event of interest was subject to competing risks, because BOT initiation may be precluded by stopping OAD therapy or by switching to insulin alone. We therefore additionally fitted a Cox model to the competing endpoint (state 2), ‘End of OAD therapy (without prior BOT initiation)’. In addition to dropouts, patients neither observed to initiate BOT nor stop OAD therapy up to October 31^st^, 2014 were treated as right-censored observations. Figure 1 gives a multistate description of the model that was used.

For the present analysis, it was not possible to also model the occurrence of ‘death (while on OAD and without initiation of BOT)’ due to sparse mortality information within the DIVE registry (only 71 deaths were recorded, cf. Fig. 2). All results were essentially comparable to a sensitivity analysis including only patients with complete covariate information (Additional file 3: Table S3). Data input, processing, management, and analyses were conducted by using R, Version 3.1.1.

## Results

### Patient characteristics at study entry

Out of the more than 130,000 patients with diabetes registered within the DIVE database, 31,008 met the inclusion criteria for the present analysis (Fig. 2). Of these, 6705 individuals (21.6 %) were observed to initiate BOT. Patient characteristics at study entry are given in Tables 1 and 2. In terms of laboratory values, the average HbA1c level was 7.5 % (SD: 1.2), with average FPG and PPG levels being 8.1 mmol/l (SD: 1.5) and 9.5 mmol/l (SD: 4.7). Note that the latter two parameters were available for only 21.4 and 14.2 % of patients (Additional file 2: Table S2).

**Table 1:** Characteristics of patients with or without observed initiation of BOT (continuous variables)

Covariate	Total population	BOT(observed)	BOT(not observed)	MD (95 % CI)
	(*N* = 31,008)	(*N* = 6,705)	(*N* = 24,303)	
	Mean (SD)	Mean (SD)	Mean (SD)	
Age (years)	62.4 (3.5)	62.4 (3.4)	62.5 (3.5)	−0.05 (−0.36, 0.26)
BMI (kg/m^2^)	32.4 (2.6)	33.0 (2.5)	32.2 (2.6)	0.7 (0.52, 0.93)
Weight (kg)	93.2 (4.6)	95.2 (4.6)	92.6 (4.6)	2.6 (1.9, 3.23)
Diabetes duration (years)	8.2 (2.7)	10.6 (2.8)	7.5 (2.7)	3.1 (2.86, 3.31)
HbA1c (%)	7.5 (1.2)	8.0 (1.2)	7.4 (1.2)	0.6 (0.55, 0.65)
FPG (mmol/l)	8.1 (1.5)	8.7 (1.6)	8.0 (1.5)	0.7 (0.58, 0.78)
PPG (mmol/l)^a^	9.5 (4.7)	10.8 (4.3)	9.1 (4.7)	0.9 (0.70, 1.14)

Legend: *BOT* basal supported oral therapy, *MD* mean difference, *BMI* body mass index, *HbA1c* glycated haemoglobin, *FPG* fasting plasma glucose, *PPG* postprandial plasma glucose. Confidence intervals (CIs) for the mean difference were derived by Welch’s t-tests

^a^based on the available data: *N* = 7,382 (Total), 1,635 (BOT(observed)), 5,747 (BOT(not observed))

**Table 2:** Characteristics of patients with or without observed initiation of BOT (categorical variables)

Covariate	Total population	BOT(observed)	BOT(not observed)	*p*-value
	(*N* = 31,008)	(*N* = 6,705)	(*N* = 24,303)	
	*n* (%)	*n* (%)	*n* (%)	
Gender				
Male	16,728 (53.9)	3,698 (55.2)	13,030 (53.6)	0.026
Female	14,280 (46.1)	3,007 (44.8)	11,273 (46.4)	
Microvascular diseases^a^				
Yes	10,851 (35.0)	2,643 (39.4)	8,208 (33.8)	<0.0001
No	20,157 (65.0)	4,062 (60.6)	16,095 (66.2)	
Macrovascular diseases				
Yes	7,377 (23.8)	1,711 (25.5)	5,666 (23.3)	<0.001
No	23,631 (76.2)	4,994 (74.5)	18,637 (76.7)	
Hypoglycaemia				
Yes	86 (0.3)	36 (0.5)	50 (0.2)	<0.0001
No	30,922 (99.7)	6,669 (99.5)	24,253 (99.8)	

Legend: *BOT* basal supported oral therapy. Test for independence between groups by means of Fisher’s exact tests

^a^Patient and/or physician reported data

### Impact of demographics on treatment regimen

Adjusted hazard ratios suggested that a higher BMI (HR: 1.012; 95 % CI: 1.007 to 1.017; Table 3) and longer diabetes duration (HR: 1.046; 95 % CI: 1.043 to 1.049) significantly increased the risk of BOT initiation, although the HRs are close to one. In contrast, female gender reduced the risk by about 5 % (HR: 0.950; 95 % CI: 0.903 to 0.998). These results are in accordance with the purely descriptive analyses (Tables 1 and 2). However, while a non-significant mean difference in age of −0.05 years (95 % CI: −0.36 to 0.25; Table 1) was found between the groups, the more precise Cox regression indicated that age did in fact have an effect. The multivariable adjusted HR of 0.993 (0.991 to 0.996; Table 3) showed a risk reduction for older individuals. This effect was reinforced by the HR for ending OAD therapy, where an increased risk was associated with an older age (HR: 1.003, 95 % CI: 1.001 to 1.005).

**Table 3:** Predictors of switch to BOT: Univariate and multivariate (pooled) HRs and corresponding lower and upper bounds of the 95 % CIs

	Endpoint	
	BOT	OAD-End
	(*N* = 6,705)	(*N* = 10,547)
Univariate		
Age	1.000 (0.998,1.002)	1.002 (1.001,1.004)
BMI	1.016 (1.012,1.020)	1.006 (1.004,1.010)
Diabetes duration	1.044 (1.041,1.047)	1.003 (0.999,1.007)
HbA1c	1.264 (1.245,1.284)	1.003 (0.984,1.022)
FPG	1.127 (1.109,1.046)	1.011 (0.999,1.023)
Gender	0.934 (0.891,0.981)	1.001 (0.963,1.040)
Microvascular diseases^a^	1.226 (1.168,1.287)	0.974 (0.936,1.014)
Macrovascular diseases^a^	1.115 (1.056,1.178)	1.131 (1.083,1.182)
Hypoglycaemia^a^	2.567 (1.850,3.563)	1.007 (0.634,1.599)
No. OADs at baseline 2 vs. 1	1.288 (1.206,1.376)	0.679 (0.636,0.725)
No. OADs at baseline 3 vs. 1	1.295 (1.054,1.592)	0.605 (0.483,0.758)
Concomitant Medication yes vs. no	1.144 (1.090,1.200)	0.834 (0.802,0.866)
Multivariate		
Age	0.993 (0.991,0.996)	1.003 (1.001,1.005)
BMI	1.012 (1.007,1.017)	1.009 (1.005,1.012)
Diabetes duration	1.046 (1.043,1.049)	1.003 (0.999,1.007)
HbA1c	1.227 (1.197,1.257)	0.999 (0.970,1.029)
FPG	1.022 (0.999,1.046)	1.014 (0.997,1.033)
Gender	0.950 (0.903,0.998)	0.995 (0.956,1.035)
Microvascular diseases^a^	1.141 (1.082,1.203)	0.947 (0.908,0.988)
Macrovascular diseases^a^	1.065 (1.004,1.130)	1.152 (1.100,1.208)
Hypoglycaemia^a^	1.708 (1.208,2.415)	0.956 (0.601,1.520)
No. OADs at baseline 2 vs. 1	1.121 (1.047,1.199)	0.680 (0.636,0.727)
No. OADs at baseline 3 vs. 1	0.981 (0.796,1.210)	0.604 (0.482,0.758)
Concomitant Medication yes vs. no	1.094 (1.042,1.149)	0.824 (0.793,0.857)

Legend: *BOT* basal supported oral therapy, *BMI* body mass index, *HbA1c* glycated haemoglobin, *FPG* fasting plasma glucose, *OAD* oral antidiabetic drug, *HR* hazard ratio. Multivariate adjustment includes all factors given in the table

^a^Patient and/or physician reported

### Impact of glycaemic control on treatment regimen

Table 3 shows that higher HbA1c levels were strongly associated with the initiation of BOT (HR: 1.227; 95 % CI: 1.197 to 1.257). This is in accordance with the observed HbA1c mean value of 8.0 % (SD: 1.2; Table 1) found for the BOT(observed) group, which was approximately 0.6 % higher than that of the group with no observed BOT initiation (95 % CI: 0.55 to 0.65). The unadjusted Cox analysis (Table 3) and the unadjusted descriptive analysis showed that FPG was significantly linked to the likelihood of BOT initiation. However, no association was detected in the multivariate analysis (HR: 1.022; 95 % CI: 0.999 to 1.046; Table 3). FPG was consistently found to have no impact on the likelihood of the competing endpoint of ending OAD therapy with no BOT initiation. Descriptive analysis found slightly higher PPG levels in patients with observed BOT initiation (MD: 0.9 mmol/l; 95 % CI: 0.7 to 1.14); however, the calculation was based on a restricted sample of patients.

### Impact of hypoglycaemia, micro-, and macrovascular diseases on treatment regimen

Only 86 individuals (0.2 %, Table 2), who had suffered episodes of hypoglycaemia during the 12 months prior to their study entries, were noted. Of these, 36 switched to BOT during the observational period. Despite this small proportion of patients, a strong association between patient reported hypoglycaemic incidences and BOT initiation was detected (HR: 1.708, 95 % CI: 1.208 to 2.416; Table 4). Further, patient or physician reported micro- (HR: 1.141; 95 % CI: 1.082 to 1.203) and/or macrovascular diseases (1.065; 95 % CI: 1.004 to 1.130) were associated with an increased risk of BOT initiation. These results are consistent with the purely descriptive results given in Table 2.

**Table 4:** Antidiabetic and concomitant pharmacotherapy with or without observed initiation of BOT

Treatment	Total Population	BOT(observed)	BOT(not observed)	*p*-value
	(*N* = 31,008)	(*N* = 6,705)	(*N* = 24,303)	
	*n* (%)	*n* (%)	*n* (%)	
Monotherapy				
Metformin	19,866 (64.1)	4,235 (63.2)	15,651 (64.4)	0.06
Sulfonylurea	4,532 (14.6)	1,012 (15.1)	3,520 (14.5)	0.21
Glucosidase inhibitors	241 (0.8)	56 (0.8)	185 (0.8)	0.53
Glitazones	158 (0.5)	33 (0.5)	125 (0.5)	0.92
Glinides	1397 (4.5)	361 (5.4)	1,036 (4.3)	<0.001
DPP-4 inhibitor	4,141 (13.4)	1,023 (15.3)	3,118 (12.8)	<0.0001
No. of OADs				<0.0001
1 OAD	26,861 (86.6)	5,564 (83.0)	21,297 (87.6)	
2 OADs	3,818 (12.3)	1,049 (15.6)	2,769 (11.4)	
≥ 3 OADs	329 (1.1)	92 (1.4)	237 (1.0)	
Concomitant medication				<0.0001
no drug	16,959 (54.7)	3,409 (50.8)	13,550 (55.8)	
1 drug	5,762 (18.6)	1,150 (17.2)	4,612 (19.0)	
2 drugs	2,697 (8.7)	566 (8.4)	2,131 (8.8)	
≥ 3 drugs	5,590 (18.0)	1,580 (23.6)	4,010 (16.5)	

Legend: *BOT* basal supported oral therapy, *DPP* dipeptidyl peptidase, *OAD* oral antidiabetic drug. Test for independence between groups by means of Fisher’s exact test

### Impact of OAD therapy on treatment regimen

A total of 86.6 % of patients were receiving a single OAD at study entry, the most common being metformin (64.1 %), sulfonylureas (14.6 %), or dipeptidyl peptidase-4 (DPP-4) inhibitors (13.4 %; Table 4). In contrast to all other compounds, Welch’s t-tests only detected significant associations for glinides and DPP-4 inhibitors (*p* < 0.001 and *p* < 0.0001, respectively). As can be seen in Table 4, more than one prescribed OAD at study entry corresponded to an increased risk for BOT initiation compared to oral monotherapy. Specifically, two OADs at study entry increased the likelihood of BOT being initiated (HR: 1.121; 95 % CI: 1.047 to 1.199, Table 3). An impact of three OADs at study entry was only detectable if the corresponding strong risk reduction regarding the competing endpoint was taken into account (HR: 0.604; 95 % CI: 0.482 to 0.758). Around 45 % of participants received concomitant medications (Table 4). These individuals were subject to a greater risk for BOT initiation (HR: 1.094, 95 % CI: 1.042 to 1.149; Table 3). Note that the direct effects corresponding to two OADs and concomitant medications were further (indirectly) increased because the risk of the competing endpoint was decreased.

## Discussion

Out of the 31,008 patients, who fulfilled the inclusion criteria in the registry, more than 20 % initiated BOT during the observational period. This value was higher than that reported by Kostev et al. (12.8 %), who also investigated initiation of BOT in patients being treated with OADs [13]. In accordance with that investigation, the present Cox analysis also found that younger age was associated with addition of basal insulin treatment. One potential reason for this is that younger age at diagnosis can indicate more severe disease, which may favour treatment with the combination of OADs and insulin. Kostev et al. [13] also found a risk increase in women; however, the present study showed the opposite effect. Higher BMI was also found to be associated with the initiation of BOT. This factor has been previously shown to be predictive for a need for multiple antidiabetic therapies for achieving target blood glucose levels [1]. Furthermore, the lower dosage of insulin generally required when used in conjunction with OADs in comparison to when used alone may limit weight gain, which would be especially beneficial in overweight patients [9]. In the present investigation, the risk for BOT initiation was found to be increased for patients with longer diabetes durations at study entry. In addition to the progressive loss of β-cell function that would have occurred over time, these patients are likely to have experienced more extensive periods of inadequate HbA1c levels, resulting in a need to intensify treatment with the addition of insulin to the oral therapy. Moreover, prolonged attempts to identify an effective oral treatment regimen may have failed, while a clinician could perceive benefits to continuing with such a strategy in patients with a more recent diagnosis.

The mean HbA1c level at study entry was found to be higher for the group of patients that switched to BOT. The observed mean value of 8.0 % is close to levels that have been stated to correspond to the point at which oral therapy has failed and insulin initiation is advised (≥8 % [20], ≥7.5 % [4, 5]). Patients not observed to initiate BOT had an average HbA1c level closer to the <7 % generally considered to be the target value for patients with type-2 diabetes [3]. In accordance with this result, descriptive analyses demonstrated higher FPG and PPG levels in the BOT(observed) group. Multivariable adjusted HRs supported HbA1c as a strong predictor for BOT. These data indicated that patients with poorer glycaemic control were more likely to switch to BOT during the observational period, which is in agreement with a previous study [13].

The presence of micro- and macrovascular diseases was found to be highly indicative of BOT initiation. It is possible that the occurrence of such events in patients being treated with OADs would encourage a physician to alter the antidiabetic therapy in order to improve glycaemic control.

The patients enrolled in the registry were prescribed a wide range of OADs either as monotherapy or in different combinations, at the discretion of the treating physician. Interestingly, descriptive analysis indicated that the use of glinides or DPP-4 inhibitors was associated with a higher risk of BOT initiation. This may merely be a further indication of the greater risk of BOT associated with poorer glycaemic control. We found that metformin use was not associated with a higher likelihood of BOT initiation, which might be indicative of physicians preferring to try an alternative oral regime before incorporating insulin. We also found that sulfonylurea use had no impact on risk of BOT initiation. The combination of metformin and a sulfonylurea is a well-established second line treatment; however, if this does not achieve adequate glycaemic control, the most suitable option for therapy intensification is unclear. Whilst guidelines suggest insulin should be initiated if an HbA1c level of ≤7.5 % (58 mmol/mol) cannot be maintained by using oral medication alone [21], there is often significant hesitation on the part of the physician to introduce such therapy [4, 5, 10, 12]. It is therefore possible that for patients receiving a sulfonylurea, there is a comparable chance of BOT initiation and change in oral therapy.

On the other hand, the more recently introduced glinides and DPP-4 inhibitors are less likely to be used as monotherapies, and at the time that the DIVE registry was established were generally only used after failure of metformin and/or sulfonylureas [2, 21]. Inadequate glycaemic control by OAD treatments including multiple agents would indicate a necessity for insulin administration, often in the form of BOT [8]. Furthermore, both glinides and DPP-4 inhibitors are compatible with insulin therapy, and the combinations have been shown to be highly effective for achieving glycaemic control [22, 23].

The prescription of more than one OAD was also indicative of conversion to BOT, in agreement with the study by Kostev et al. [13]. It is likely that treatment intensification for patients receiving a single OAD at baseline would proceed via prescription of a second oral drug rather than insulin. This is in agreement with our data showing that metformin use was not indicative of BOT initiation. As this agent is usually the first line pharmacological therapy for patients with type-2 diabetes, the majority of those receiving a single OAD at baseline would be taking metformin.

The presence of concomitant medications was also found to be a negatively associated with the initiation of BOT. This is in accordance with the identified incidence of micro- and macrovascular disease in patients that switched to BOT, as comorbidities generally correlate with number of drugs being administered.

Some limitations affected the certainty of the present findings. 1) The determination of OAD and BOT periods was based on preliminary assumptions. Periods that were less than 6 months apart were merged into one period. Furthermore, missing information about the end of treatments were right-censored 6 months after the last available medication record because prescriptions are usually made for a period of 3 months, and rarely beyond 6 months. However, these choices were clinically defensible as there may have been cases where antidiabetic treatment was prescribed by other physicians, and therefore not recorded. 2) Medication prescriptions before the start of DIVE were generally unknown or assumed to be inaccurate. For persons with recent diabetes diagnosis dates, the OAD initiation was based on up-to-date registry data. In contrast, the OAD initiation for persons being not recently diagnosed was only determinable by means of the recorded prescription data. 3) The present investigation did not impute missing PPG levels as these were absent for such a high proportion of patients (75 %); therefore, information was expected to be too speculative. For this reason, analysis of PPG was performed using only descriptive measures, with further investigation required to determine any potential impact of this variable. 4) Hypoglycaemic events and clinical diagnoses regarding comorbidities were recorded based on recollection by the patient rather than medical records; therefore, some inaccuracies may have been introduced into the analysis. Nevertheless, the information was assumed to be sufficiently reliable as events were likely to have been severe in nature. 5) All analyses were conducted by means of censoring possible competing death events as the DIVE registry contained a very small number of deaths. Nevertheless, the present approach produced valid HR estimates (Competing risks and multistate models with R), but probability predictions and individual prediction models would require additional mortality information.

The major strength of the present investigation was the high number of patients and the quality of the data. The register-based dataset provided a high number of eligible individuals and a reasonable number of events. This allowed the computation of point estimates with very small confidence intervals. Missing information for covariates led not to exclusion, but was addressed by means of a multiple imputation approach. The competing risks model allowed for left-truncated data, where individuals entered the risk set at different time points. However, the analysis required the use of covariate information collected at study entry and, thus, not necessarily at OAD initiation. The temporal development of the medical problem was adequately taken into account and it was not conditional on future events. Simultaneous analysis of the competing endpoint also led to a better understanding of the underlying processes [24], and enabled the detection of indirect covariate effects on the risk of BOT initiation.

## Conclusions

Analysis of the DIVE registry has resulted in the identification of a number of factors that may be predictive for the initiation of BOT for type-2 diabetes patients initially prescribed one or more OADs. Poor glycaemic control, the presence of vascular comorbidities and concomitant medications, and a greater number of OADs were all detected to increase the risk of a switch to BOT. Female gender and younger age showed protective properties. The close monitoring of patients displaying these characteristics may help to identify those for whom oral therapy alone is unlikely to be sufficient, and therefore who might benefit from early addition of insulin therapy to their treatment regimen.

## Additional files

Additional file 1: Table S1. Concomitant pharmacotherapy: ATC indices*. Legend: DDP-4, dipeptidyl peptidase-4. *World Health Organisation Collaborating Centre for Drugs Statistics Methodology. (DOCX 14 kb) Additional file 2: Table S2. Absolute Number of Missing Values and Relative Proportion w.r.t. total study population. (DOCX 14 kb) Additional file 3: Table S3. Predictors of switch to BOT: Multivariate HRs and corresponding lower and upper bounds of the 95 % CIs including only patients with complete covariate information. (DOCX 16 kb)

## Abbreviations

BMI: Body mass index
BOT: Basal supported oral therapy
CI: Confidence intervals
DIVE: DIabetes Versorgungs-Evaluation
DPP-4: Dipeptidyl peptidase-4
FPG: Fasting plasma glucose
GLP-1: Glucagon-like peptide-1
HbA1c: Glycated haemoglobin A1c
HR: Hazard ratio
MAR: Missing at random
MD: Mean differences
NPH: Neutral protamine hagedorn
OAD: Oral antidiabetic drugs
OR: Odds ratios
PPG: Postprandial plasma glucose
PRIND: Prolonged reversible ischaemic neurologic deficit
SD: Standard deviation
TIA: Transient ischaemic attack
WHO: World Health Organization
