# Supplementary material for: Treatment intensification using long-acting insulin –predictors of future basal insulin supported oral therapy in the DIVE registry
Source: BMC Endocr Disord. 2015 Oct 7;15:54. doi: 10.1186/s12902-015-0051-0 (PMC4597397; doi:10.1186/s12902-015-0051-0)
Supplement: Additional file 1: Table S1. — Concomitant pharmacotherapy: ATC indices*. Legend: DDP-4, dipeptidyl peptidase-4. *World Health Organisation Collaborating Centre for Drugs Statistics Methodology. (DOCX 14 kb) [file 12902_2015_51_MOESM1_ESM.docx]

## Additional table 1 - Concomitant pharmacotherapy: ATC indices*

| **Medication** | **ATC index** |
| --- | --- |
| Metformin | A10BA02 |
| Sulfonylurea | A10BB |
| Glucosodase inhibitors | A10BF |
| Glinides | A10BX02, A10BX03, A10BX08 |
| Glitazones | A10BG |
| DPP-4 inhibitors | A10BH |
| Comb. Metformin | A10BD |

Legend: DDP-4, dipeptidyl peptidase-4. *World Health Organisation Collaborating Centre for Drugs Statistics Methodology.
