# Supplementary material for: Treatment intensification using long-acting insulin –predictors of future basal insulin supported oral therapy in the DIVE registry
Source: BMC Endocr Disord. 2015 Oct 7;15:54. doi: 10.1186/s12902-015-0051-0 (PMC4597397; doi:10.1186/s12902-015-0051-0)
Supplement: Additional file 2: Table S2. — Absolute Number of Missing Values and Relative Proportion w.r.t. total study population. (DOCX 14 kb) [file 12902_2015_51_MOESM2_ESM.docx]

## Additional table 2 - Absolute Number of Missing Values and Relative Proportion w.r.t. total study population

| **Covariate** | **No. of NAs (%)** |
| --- | --- |
| Diabetes duration | 7,852 (25.3) |
| Height | 7,826 (25.3) |
| Weight | 9,383 (30.3) |
| HbA1c | 11,773 (38.0) |
| FBG | 21,660 (69.9) |
| PPG | 23,626 (76.2) |
