# Supplementary material for: Treatment intensification using long-acting insulin –predictors of future basal insulin supported oral therapy in the DIVE registry
Source: BMC Endocr Disord. 2015 Oct 7;15:54. doi: 10.1186/s12902-015-0051-0 (PMC4597397; doi:10.1186/s12902-015-0051-0)
Supplement: Additional file 3: Table S3. — Predictors of switch to BOT: Multivariate HRs and corresponding lower and upper bounds of the 95 % CIs including only patients with complete covariate information. (DOCX 16 kb) [file 12902_2015_51_MOESM3_ESM.docx]

## Additional Table 3 - Predictors of switch to BOT: Multivariate HRs and corresponding lower and upper bounds of the 95% CIs including only patients with complete covariate information

| **Covariate** | **Endpoint** | |
| --- | --- | --- |
|  | **BOT**  **(N = 1,122)** | **OAD-End**  **(N = 2,358)** |
| Age | 0.995 (0.990,1.001) | 1.004 (0.999,1.009) |
| BMI | **1.020** (1.011,1.030) | 1.009 (0.999,1.019) |
| Diabetes duration | **1.058** (1.051,1.064) | 1.001 (0.992,1.010) |
| HbA1c | **1.297** (1.240,1.356) | 0.967 (0.911,1.026) |
| FPG | **1.062** (1.031,1.095) | 0.972 (0.940,1.007) |
| Gender | 0.961 (0.849,1.088) | 1.121 (0.994,1.264) |
| Patient reported microvascular diseases | 1.102 (0.968,1.255) | 0.976 (0.856,1.113) |
| Patient reported macrovascular diseases | **0.851** (0.725,1.000) | **1.352** (1.171,1.561) |
| Patient reported hypoglycaemia | **2.345** (1.249,4.488) | 0.479 (0.067,3.419) |
| No. OADs at baseline 2 vs. 1 | 1.146 (0.977,1.344) | 0.833 (0.684,1.015) |
| No. OADs at baseline 3 vs. 1 | 1.454 (0.973,2.173) | 0.637 (0.302,0.1.343) |
| Concomitant Medication yes vs. no | 1.105 (0.977,1.250) | **0.833** (0.739,0.941) |

Legend: BOT, basal supported oral therapy; BMI, body mass index; HbA1c, glycated haemoglobin, FPG, fasting plasma glucose; OAD, oral antidiabetic drug; HR, hazard ratio. Bold format indicates a significant impact risk for the event of interest. Multivariate adjustment includes all factors given in the table.
